# Cellular electron cryo tomography and *in situ* sub-volume averaging reveal the context of microtubule-based processes

**DOI:** 10.1016/j.jsb.2016.06.024

**Published:** 2017-02

**Authors:** Michael Grange, Daven Vasishtan, Kay Grünewald

**Affiliations:** Oxford Particle Imaging Centre, Division of Structural Biology, Wellcome Trust Centre for Human Genetics, University of Oxford, Oxford, OX3 7BN, United Kingdom

**Keywords:** Electron cryo-tomography, Cellular architecture, Sub-volume averaging, Microtubules, Cytoskeleton, Dynein, Adenovirus, Electron cryo-microscopy, Virus trafficking, Viral entry, Retrograde transport, Endocytosis, *in situ* structure determination

## Abstract

Electron cryo-tomography (cryoET) is currently the only technique that allows the direct observation of proteins in their native cellular environment. Sub-volume averaging of electron tomograms offers a route to increase the signal-to-noise of repetitive biological structures, such improving the information content and interpretability of tomograms. We discuss the potential for sub-volume averaging in highlighting and investigating specific processes *in situ*, focusing on microtubule structure and viral infection. We show that (i) *in situ* sub-volume averaging from single tomograms can guide and complement segmentation of biological features, (ii) the *in situ* determination of the structure of individual viruses is possible as they infect a cell, and (iii) novel, transient processes can be imaged with high levels of detail.

## 1 Introduction

Electron cryo tomography (cryoET) is a technique used to reconstruct 3-dimensional structures of biological samples in their native, frozen hydrated state. By taking a tilt series of images of vitrified cells (or other relevant specimens) in an electron microscope it becomes possible to visualize unique pleomorphic events, structures with short-lived and transient states, and large macromolecular organisations (e.g. lattices) that would be otherwise very difficult to reconstitute *in vitro* (Asano et al., 2015, Brandt et al., 2010, Hagen et al., 2015, Schur et al., 2015, Zeev-Ben-Mordehai et al., 2016). Furthermore, repetitive or frequently occurring molecular structures within these pleomorphic objects can be averaged together to create higher resolution maps, a technique generally referred to as sub-tomogram averaging.

Recent technical developments in electron cryo microscopy (cryoEM) have resulted in the ability to image and collect data of vitrified specimens with unprecedented signal-to-noise ratios, allowing the analysis of biological structures and features at a much higher level of detail than previously attainable. These developments include better zero-loss energy filters and direct electron detectors, both of which have vastly improved the quality of tomographic data acquisition (Koster et al., 1997, Li et al., 2013, McMullan et al., 2014), and led to the high-resolution determination of novel macromolecular complexes and viruses, as well as the structural determination of pleomorphic complexes and the *in situ* architecture of cells (Asano et al., 2015, Bartesaghi et al., 2015, Fukuda et al., 2015, Mahamid et al., 2016, Matthies et al., 2016, Schur et al., 2015, Sirohi et al., 2016, Zeev-Ben-Mordehai et al., 2016).

This move to analyze native cellular structures *in situ* has come concomitantly with a push towards the development of technologies such as Zernike phase plates, and more recently Volta phase plates (VPP) (Danev et al., 2014, Murata et al., 2010), that improve the image contrast. These improvements further include specimen thinning methods mitigating some of the detrimental impact of thick specimens – cells are typically too thick to image through standard methods, as the penetration limit of electrons through a frozen-hydrated specimen at 300 keV is around 1 μm (Baumeister, 2002) – and allow us to use cryoET to image processes anywhere in the interior of cells (Al-Amoudi et al., 2004, Rigort et al., 2012). However, such techniques are still limited and challenging to implement technically.

Alternatively, it is also possible to avoid this thickness limit by focusing attention to the cell periphery. These areas of the cell are generally thin enough to be successfully imaged, and are accessible directly after plunge freezing such offering a much more widely and routinely used route of specimen preparation for many structural and cell biologists. Understanding the cell periphery is crucial to comprehending many biological mechanisms. These include, but are not limited to, cell signaling, translation, exocytosis, endocytosis, pathogen entry and egress and cytoskeletal organization and trafficking (Brandt et al., 2010, Cyrklaff et al., 2007, Dodonova et al., 2015, Ibiricu et al., 2011).

**Fig. 1:**
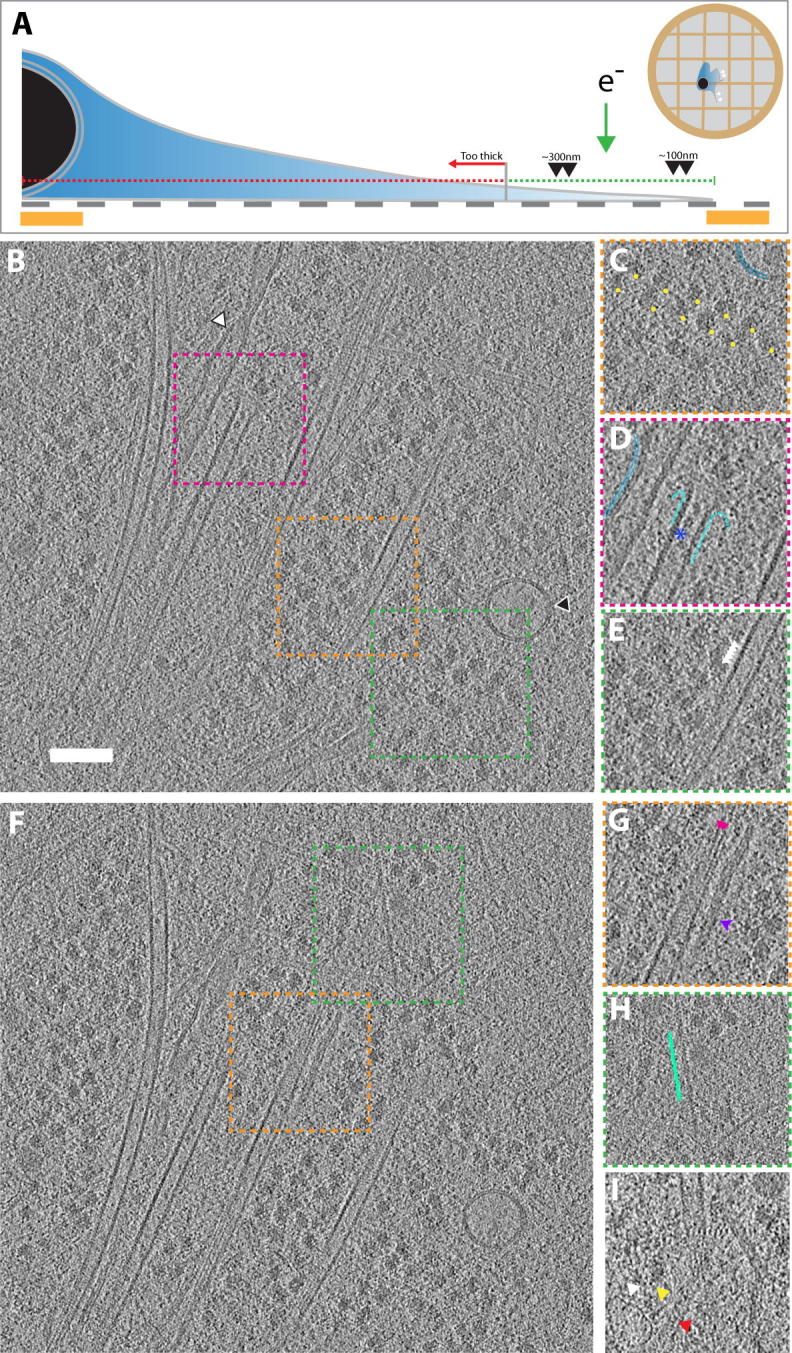
CryoET of a peripheral region of an adherent U87MG neuronal cell line imaged using direct electron detection. (A) Schematic representation of an adherent cell grown on a holey carbon-coated gold grid. Red line indicates regions too thick for cryoET imaging. Area marked by green line indicates typical regions of the cell accessible to imaging by cryoET. (B) Slice through tomogram at the periphery of the U87MG cell, containing a number of cellular features such as (C) polysomes, (D) a de-polymerising microtubule in proximity to the endoplasmic reticulum with microtubule luminal bodies (blue asterisk), and (E) 4 nm tubulin spacing of the microtubule. White and black arrows in (B) represent the endoplasmic reticulum and a lysosome, respectively. (F) Another slice through the same tomogram, showing the diversity of cytoskeletal elements visible in a snapshot of the cell periphery; (G) microtubule protofilaments (pink arrows) (top-view), intermediate filaments (purple arrow) and (H) F-actin (green). (I) Vault ribonucleoprotein cage (white arrow) and a putative cytoskeletal motor, dynein (yellow arrow), engaged with a microtubule (red arrow), cf. also Fig. S2. Squares outlined by colored dashed lines in (B) and (F) indicate positions of areas in (C–E) and (G–H), respectively. Scale bar; 100 nm. Thickness of the tomographic slices is 4.22 Å for all panels.

In this work, we assess what can be achieved by targeting the cellular periphery of whole intact non-thinned cells in individual tomograms, particularly through sub-volume averaging and the localization of rare events (Fig. 1A). We discuss the opportunities that novel developments offer to structural cell biologists when using direct electron detectors, especially when combined with an energy filter. Our assessment shows that the majority of information to determine usable and contextual structures is contained within the tomogram. As examples we reveal a range of regular structures that are discernible throughout the tomograms and assess their roles and interaction in the course of viral entry.

## 2 Materials and methods

### 2.1 Cell culture and plunge freezing

U87MG cells and U2OS cells were seeded and grown on poly-l-lysine-coated gold mesh carbon coated holey carbon grids (Electron microscopy sciences) in Dulbecco’s modified eagle medium (DMEM) (Gibco) supplemented with 10% fetal bovine serum. The cells were grown to approximately 60–80% confluency, or until 1–2 cells per grid square. The grids were supported in 2×9-well μ-slide co-culture dishes (Ibidi). Adenovirus particles were cultured and isolated as has been described previously (Wodrich et al., 2010). For infection of U2OS cells with adenovirus, 4 μl of adenovirus was mixed in 200 μl of DMEM. 50 μl was applied to each grid and incubated with the cells for 10–20mins before plunge freezing. Grids were plunge frozen in an ethane/propane mix, cooled to liquid nitrogen temperature using a manual blotting apparatus, blotted from the backside of the grid for between 2 and 4 s.

### 2.2 Electron microscopy

Tomograms were acquired using a Tecnai TF30 “Polara” Microscope (FEI, Eindhoven) operating at 300 keV. The microscope was equipped with a Gatan Imaging Filter “Quantum” energy filter operated in zero-loss mode with a 20 eV wide slit, and a post energy filter mounted K2 Summit direct electron detector (Gatan, Inc.) Data were acquired in counting mode, with aligned frames produced ‘on the fly’, using the combined filter for image alignment in Digital Micrograph (Gatan, Inc.). Dose, pixel size, magnification and tilt angle information are provided in Table S1. Data were acquired using SerialEM (Boulder, Colorado) (Mastronarde, 2005).

### 2.3 Tomogram reconstruction, segmentation and subvolume averaging

Tilt series outputted from the microscope were aligned and reconstructed using the IMOD software package (Kremer et al., 1996). CTF correction, where applicable, was also performed using IMOD. A global defocus was determined for the whole tomogram, which was then used to correct individual tilts in the tilt series, taking into account the change in eucentricity across the projection images at high tilts. Phase flipping was then performed on each tilt image and the tomogram reconstructed using this phase flipped image stack. Sub-volume averaging of microtubules was performed using PEET (also part of IMOD) (Nicastro et al., 2006). Particles were picked from tomograms binned by a factor of 4, using scripts based on TEMPy (Farabella et al., 2015) that evenly sampled at 4 nm intervals (based on the size of one tubulin monomer) through a straight line defined by the user, with each particle oriented parallel to the line. These particles were then used for iterative sub-volume refinement; with further refinement applied on factor 2 binned and unbinned tomograms. Custom scripts were used to relate reconstructions between the binning levels of the tomograms to utilize the respective Euler angles and translational information. Sub-volume averages were acquired at binning factors of 4,2 and 1 for the U87MG tomogram (final pixel size = 4.22 Å unbinned), and binning factors of 4 and 2 for the U2OS tomogram (final pixel size = 4.6 Å at binning factor 2). Symmetrisation of the microtubules was performed using custom scripts, and utilized the prior knowledge from the unsymmetrised structure that the microtubules contained 13 protofilaments, with 4 nm tubulin dimer spacing (on average).

The picked microtubule particles were split in half at random prior to sub-volume averaging and each half dataset independently averaged during refinement, including symmetrisation. Each run of sub-volume averaging at every binning level for the reconstruction was performed three times: one on the first random half dataset, the second on the second half and a third on the combined data. In the case of the U2OS tomogram, at the end of refinement, particles with a cross correlation of less than 0.2 were excluded. In the case of both tomograms, if any particles had centres within 4 nm of each other, the particle with the lower cross correlation was excluded. Resolution determination was assessed through FSC at 0.5 and 0.143 between the two half set reconstructions (the ‘gold standard’ criterion). Structures shown in the paper are from the combined dataset, filtered to the resolution cut-off determined by FSC0.143 between the two half sets. Curves were determined using a number of different masks, specifically: no mask, the final microtubule structure binarised at a noiseless threshold and extended with a soft edge (threshold mask), or a hard edge cylinder or soft edge cylinder of approximate microtubule diameter (Fig. 3F). FSC curves and masks for “gold-standard” FSC determination were generated using EMAN2 (Tang et al., 2007).

Refinement of the adenovirus structure was performed using PEET, utilising an option to not produce a new map after each iteration of refinement. The adenovirus reference structure (EMDB-1574) was filtered to 60 Å prior to alignment. The determined position and orientation of the picked particle was then used to symmetrize it and produce a reconstruction. We also produced a “back-plotted” tomogram, where the virus particles in the tomographic volume were substituted with the reconstruction using a custom script that utilized the translation offsets and Euler angles for each given particle position.

Segmentation and representation of tomograms (including back-plotted tomograms) was performed and viewed using the visualization software Amira (FEI, Eindhoven). Structures resulting from sub-volume averaging and backplotted coordinate models were visualized using Chimera (UCSF) (Pettersen et al., 2004).

## 3 Results and discussion

### 3.1 Whole cell electron cryo tomography of the cellular periphery using direct electron detectors

Using a 300 keV FEG electron microscope with post-energy filter mounted direct electron detector (Gatan K2 Summit), we first aimed to assess visually what information could be gained by following relatively regular and populous features within single tomograms of adherent human cells. We comparatively performed this analysis on two tomograms from two different cells lines: 1) U87MG cells and 2) U2OS cells in areas of ∼80–200 nm thickness (Figs. S1 and S2).

Visual inspection of tomograms is a useful first approach in analyzing and characterizing the major components (cf. its use in some notable recent studies (Fukuda et al., 2015, Mahamid et al., 2016)). We clearly observed and easily discriminated the different components of the cytoskeleton, such as intermediate filaments, F-actin and microtubules (Fig. 1B, F) due to their characteristic structural signatures. The overall level of detail in these tomograms was very high, allowing us to clearly discern, e.g. for the cytoskeletal structures, considerable sub-structural detail. For F-actin, we could observe the zig-zag nature of F-actin (Fig. 1H, green line), a ∼2.8 nm repeat feature, as well as the globular features of individual actin monomers within the filament (Fujii et al., 2010). Similarly, the intermediate filaments showed a rather smooth parallel tubular architecture slightly wider in diameter (∼10 nm) than actin filaments (∼7 nm), consistent with their tightly packed, cylindrical structure (Herrmann et al., 2007).

However, the greatest level of detail visible in the tomogram was found when considering the microtubules. Protofilaments were clearly visible in the top and bottom views of the microtubules. Furthermore, the tubulin lattice in the microtubule wall was evident with the 4 nm spacing of tubulin monomers being clearly distinguishable through top, bottom and middle slices of the microtubules (Fig. 1E, G), suggesting a high level of detail being contained within the tomogram. The high signal-to-noise ratio further allowed us to visualize more transient states in these filaments, such as a microtubule plus end in the process of disassembly (Fig. 1D) (Koning et al., 2008).

As well as elements of the cytoskeleton, the tomogram also showed less regular, more globular macromolecules. Ribosomes were often arranged into polysomes (Fig. 1C), as has been characterized previously (Brandt et al., 2010). The level of detail allowed us to differentiate the 50S and 40S subunits. A ribonucleoprotein “Vault” (Woodward et al., 2015) could be observed, in close proximity to a microtubule and potentially engaged with a dynein motor (Figs. 1I, S2). Putative AAA+ domains of a dynein motor complex as well as the respective stalk regions projecting onto a nearby microtubule are visible (Figs. 1I, S2) (Roberts et al., 2009). Vaults are ribonucleoprotein organelles that are thought to recruit and store mRNAs. This function has led to the suggestion that they act as a transport cages within the cytosol (Chugani et al., 1993). Our observation adds credit to the idea that Vaults are important in the transportation of mRNAs and other cargoes inside cells.

Further to macromolecules, various organelles could also be identified within the tomogram, such as the lysosome and the endoplasmic reticulum (Fig. 1B, arrows), with their 6–8-nm-thick bilayer distinctly visible.

### 3.2 *In situ* sub-volume averaging from a single tomogram

**Fig. 2:**
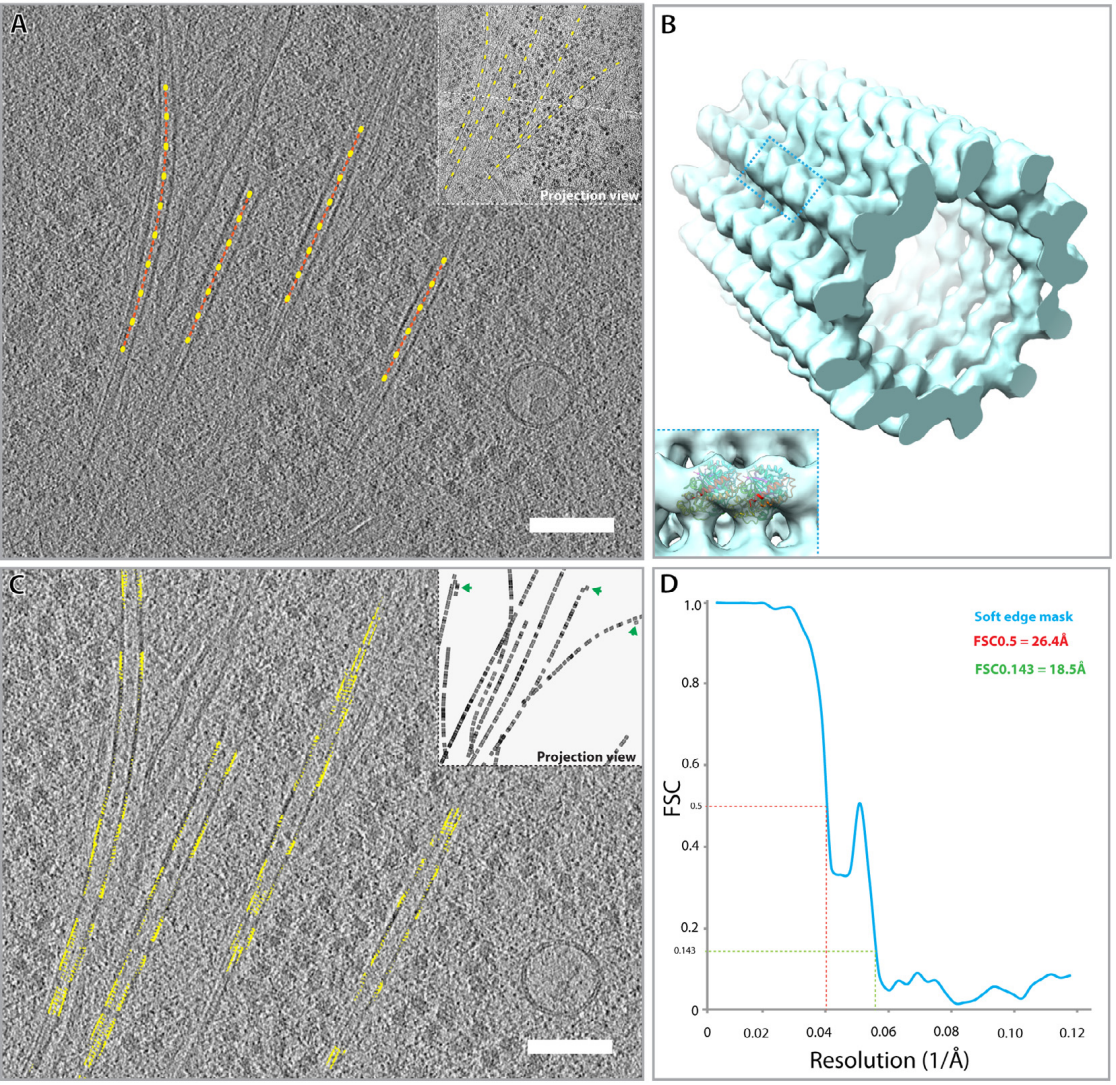
Sub-volume averaging of microtubules. (A) Schematic model of how microtubules were picked for sub-volume averaging shown in the context of a single tomographic slice and as a projection of the whole tomogram (inset). (B) Resulting sub-volume average of the microtubule from this single tomogram in (A) filtered to 18.5 Å according to the calculated gold standard FSC, at threshold 0.143. Tubulin α/β dimer (PDB: 1TUB) is shown docked into the structure (inset, blue). (C) Tomographic slice overlaid with the equivalent slice in the back-plotted tomogram and projection (inset) of the microtubule average shown in (B). Green arrows in the inset indicate outlier particles. These outliers particles are found in regions of the tomogram that may be less well aligned (i.e. edge of tomogram) and are included in the final average. (D) Gold standard FSC curve for the microtubule structure determined from the tomogram. The FSC was produced using a soft-edge cylindrical mask. Scale bar for (A) and (C) is 100 nm.

In order to assess the potential resolution achievable from this tomogram of a peripheral U87MG cell area, we performed sub-volume averaging of microtubule segments (Fig. 2). Due to the fact that the microtubules were oriented roughly perpendicular to the tilt-axis for the tomographic series, we expected that we would be able to compensate to some extent the information lost due to the CTF, as different apparent defoci of extracted volumes caused by tilting of the sample would compensate for some of the lost information. The resultant microtubule structure has 13 protofilaments, with a tubulin monomer spacing of ∼4 nm, consistent with the current understanding of the microtubule structure *in vivo* (Evans et al., 1985, Kollman et al., 2010)*.* As the sampling of the tomogram precluded the possibility of discerning the microtubule seam in these structures, we applied a 13-fold helical symmetry to the final model to produce the final structure. The gold standard Fourier Shell Correlation curve of the resultant microtubule structure (Fig. 2B) indicated a resolution of 18.5 Å at the 0.143 threshold (Fig. 2D). A prominent local peak occurred at a spatial frequency equivalent to ∼22 Å resolution (Figs. 2D and 4A). Analysis of the Fourier spectrum of the microtubule structure indicated a strong layer line at this resolution, as has been seen for other microtubule structures (Alushin et al., 2014). The calculated defocus and the nominally acquired microscope defocus differed by up to 1 μm, and accounting for inaccuracies of the defocus due to spherical aberration in the microscope, as well as the range of defoci across the tomogram, it is difficult to determine an overall single defocus. We therefore proceeded with the IMOD determined defocus (Xiong et al., 2009) as basis for comparisons.

The resulting microtubule structure is of correct size and shape to fit a crystal structure of the tubulin αβ-dimer (PDB: 1TUB) (Fig. 2B, inset). Due to the nature of tomographic data acquisition, where a range of defoci likely exist within the same dataset, the zeroes of the CTF will not be clearly visible in a sub-tomogram average if a structure that prevails throughout the field of view in a single tomogram is used for sub-volume averaging. Our analysis suggests that the sub-volume averaging performed from this single tomogram is however very likely limited in resolution by the number of particles contained within the tomogram; here 4056 overlapping sub-volumes of 25 nm × 25 nm contributed to the final average.

In the case of single particle reconstruction methods, resolution has been shown to be very sensitive to the applied electron dose (Bartesaghi et al., 2014). An element where the dose may be important in sub-tomographic averaging is when incorporating high tilts into the final sub volume average. Studies have shown that lower tilt images are the main contribution to good resolution sub-volume averages (Bartesaghi et al., 2012) and recent strategies have been developed to down weight the contribution of higher tilts (Bharat et al., 2015). However, high tilts can be very important in the reduction of back-projection artifacts from gold fiducials in tomograms, which may otherwise interfere with the interpretation of rare individual events in cellular tomograms that do not benefit from sub-volume averaging.

**Fig. 3:**
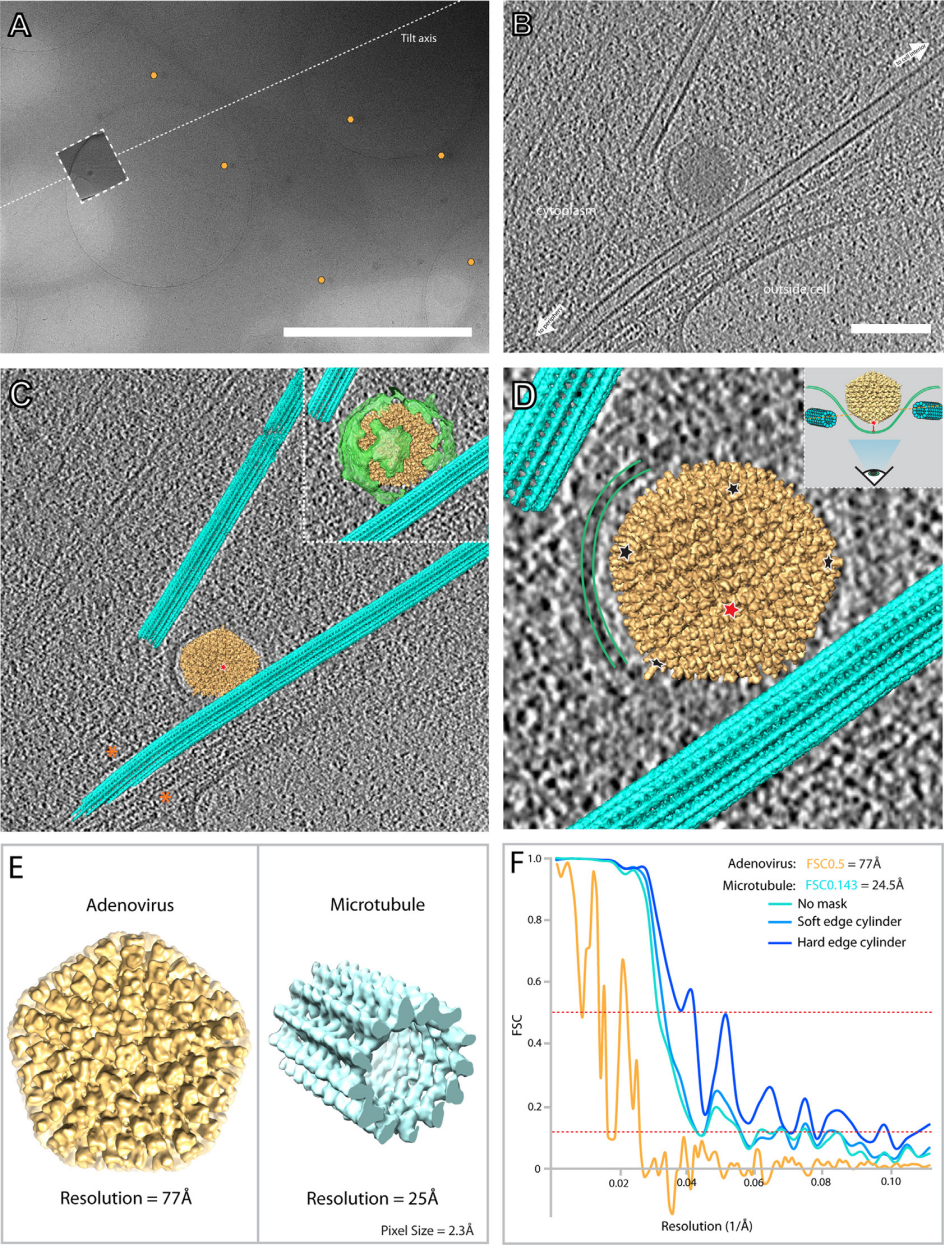
Endosomal entry of Adenoviruses as a case for structure determination *in situ*. (A) Low magnification projection image (9500×, nominal) of the field of view of an U2OS cell infected with adenoviruses (marked by yellow hexagons). The tilt axis is shown as a white dotted line. (B) High magnification (95000×, nominal) slice of a tomogram taken at the position shown by the dashed-outlined box in (A) at approx. −5.2 μm defocus. (C) Tomographic slice taken at an approximately 45° angle relative to the slice shown in (B), with the microtubule (cyan) structure and an adenovirus (yellow) structure (filtered to 20 Å – EMDB-1574) back-plotted into the tomogram. Green (inset) shows a view providing also the manually segmented surrounding endosomal membrane. Orange asterisks denote the peripheral F-actin. (D) View from below the adenovirus, showing the localization of the penton bases of the adenovirus (red and black asterisks). (E) Left: Adenovirus structure (yellow) produced from the icosahedral average of the *in situ* adenovirus determined using the calculated Euler-angles of the back plotted virus and subsequent sub-tomographic averaging. Right: microtubule structure (cyan) produced from this tomogram. Adenovirus is approx. 960 Å in diameter. Microtubule is approx. 25 nm in width. (F) FSC curves of the single adenovirus (yellow) and the microtubule average (cyan – green). The colour of the curve indicates the type of mask used in the final gold standard FSC determination of the microtubule. Resolution stated is unmasked. Scale bars are 2 μm and 100 nm for (A) and (B), respectively.

**Fig. 4:**
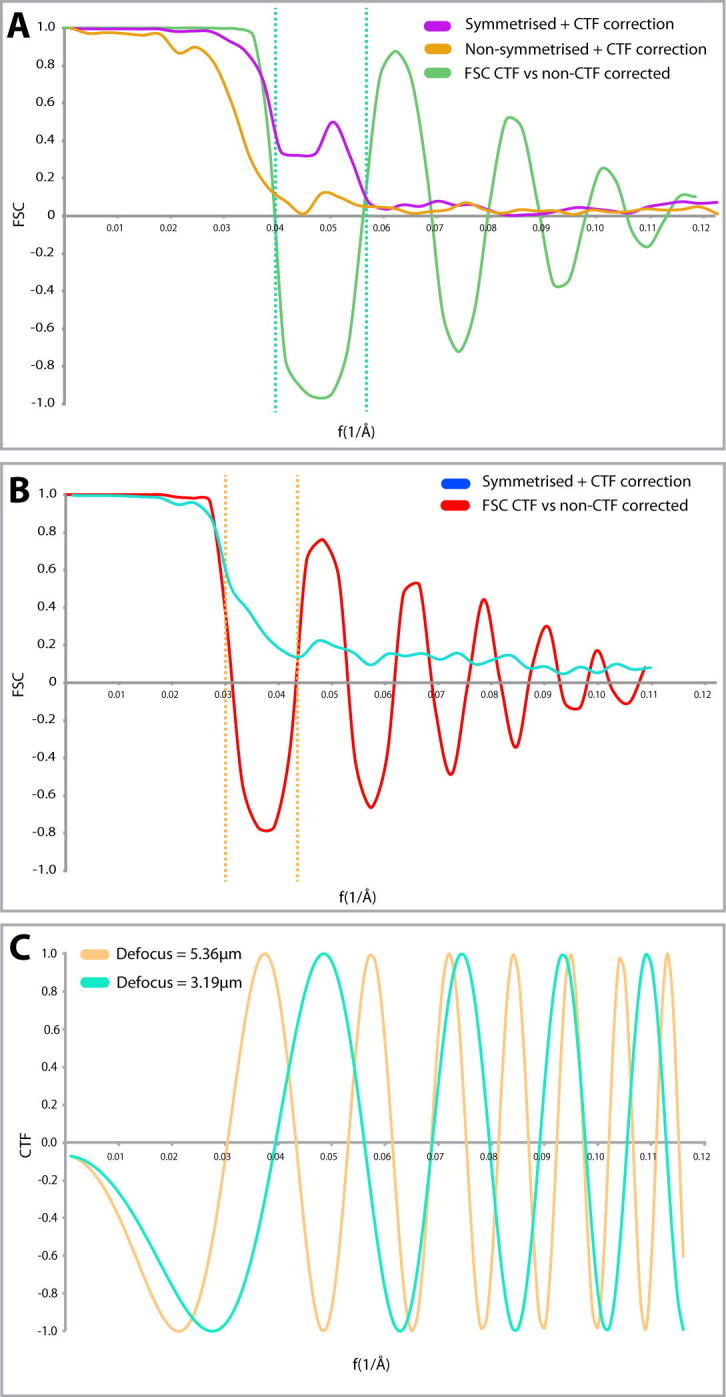
Sub-volume averaging *in situ* in a single tomogram is enough to correct for the CTF, and results in a corrected sub-volume averaged structure. (A) CTF curves of sub-volume averages produced from CTF corrected sub-volume average microtubule from the tomogram of the U87MG cell before (orange) and after (magenta) symmetrisation. The green curve represents the FSC of the non-CTF corrected average when compared to the CTF-corrected structures in both cases. This produces the expected negative correlation due to the effect of the CTF (green dotted line). (B) FSC curve of the CTF-corrected U2OS cell microtubule reconstruction (blue). FSC of the equivalent non-CTF corrected when compared to the CTF-corrected structure (red). Lack of fall-off to zero of the CTF corrected structure is due to partial alignment of noise during symmetrisation of the microtubule average after alignment. (C) CTF calculation for the determined defoci for the tomograms in (A) (cyan) and (B) (brown). Phase flipping, and the range of defoci of particles within the tomograms has compensated for the inverted contrast and node of zeroes caused due to the oscillations in the CTF. This indicates that the range of defoci within a tilt series is enough to compensate for a single nominal acquisition defocus. Masks used to produce the FSC are a soft cylindrical mask in A) and no mask in B).

Sub-volume averaging of microtubules in the U2OS cellular tomogram (Fig. 3) resulted in a microtubule structure of 25 Å. This resolution was determined without a mask as use of a mask appeared to produce an FSC at 0.143 in noisy regions of the FSC curve, as shown in Fig. 4C. Due to the higher magnification, and resultant smaller field of view, only a quarter of the number of particles could be picked (Table S1). Only particles with a cross-correlation coefficient (CCC) score above 0.2 after alignment were included in the final average. Removal of particles below this threshold removed the majority of the microtubule particles that were in the thicker regions of the tomograms (Fig. S4). A thickness gradient from ∼90 to ∼180 nm existed across this tomogram (Fig. S3). It therefore seems likely that the main contribution to the low CCC in this model is due to the increased thickness. However, it has been shown before that filamentous objects perpendicular to the tilt axis suffer from distortions that may affect their potential to align them accurately (Mastronarde, 1997). Interestingly, these microtubules also aligned parallel to the tilt axis, suggesting the alignment may possibly be dependent on the tilt axis. The reduced signal to noise and low cross correlation, and subsequent removal of bad particles most likely contributed to the difficulty in resolution determination during masking.

In the case of this second microtubule sub-volume average structure, the fall-off in cross correlation between the two independent halves of the dataset did not fully reach zero (Fig. 3F), suggesting that there is inherent noise in this structure, possibly introduced by particles from the thicker regions of the sample. This is further validated by comparison with the non-CTF corrected FSC curve, which was generated by replacing the CTF-corrected tomogram with its non-CTF corrected equivalent, without further alignment of the particles. Since this changes the noise floor, the noise correlation disappears, as evidenced by the near zero values of the non-CTF corrected FSC curve at higher resolutions (Fig. S5B).

### 3.3 Backplotting of sub-volume average structure into the tomogram results in a 3-dimensional context for structural interaction

The ability to gain biological insights from cryoET data depends intrinsically on our ability to mine and detect the *in situ* context of our biological feature of interest. To allow better probing the relative localization of features in the cell, we used the refined positions and orientations of the individual particles contributing to the average. The microtubule average was back plotted (see Section 2) into the raw tomogram, which are of comparably low signal-to-noise and thus make it difficult to directly visualize a molecular interaction. In this instance, we applied two methods: a) direct back plotting of the sub-tomogram average structure using the positions and orientations determined during alignment, and b) refining the orientation of a known structural feature into the respective position in the tomogram, in this case performed for an adenovirus virus capsid.

Sub-volume averaging of the microtubule was performed as described above. For the adenovirus re-orientation, we used PEET to fit a previously determined viral structure (EMDB ID 1574, (Fabry et al., 2008)) into our tomographic viral feature. The orientation of this structure was then subsequently refined and placed into the tomogram according to the determined orientation. The fitting of the structure was then compared to the original tomogram, to verify and compare whether the adenovirus densities overlapped with features in respective slices through the back-plotted volume.

To further validate the accuracy of the alignment of the EMDB-structure to the adenovirus contained within the tomogram, we performed icosahedral averaging of the single adenovirus within the tomogram, according to the Euler angles determined during the alignment of the EMDB-map. The resultant symmetrically averaged structure yielded a structure of 77 Å, when compared to a low-pass filtered map of EMDB-map at 20 Å (Fig. 3F). The icosahedral average of the single virus before and after orientation refinement is presented in Fig. S6. The symmetrized adenovirus structure allowed for direct visualization of the trimeric hexon proteins on the virus surface, as well as of the 12 penton bases. Icosahedral symmetrisation of the virus in the absence of any refinement yielded no discernible structure (Fig. S6) indicative of a lack of symmetry axis orientation and possible position inaccuracy. This analysis shows that the information contained in our tomogram was able to produce correlative signal to 77 Å (Fig. 3E, F) from only 60 copies of an asymmetric unit, with the noisy FSC curve being indicative of particle number based limitation and even further information content. The demonstrated potential for the differential structural determination of sub-populations of virus and protein structures *in situ* provides promising and exciting possibilities for dissecting dynamic processes and subtle structural changes *in situ*.

The resultant “segmented” tomogram (Fig. 3C, D), allowed for directly viewing the 3-dimensional spatial organization of the microtubule network of an adenovirus undergoing viral entry. We were able to observe the topology and specific orientation of an adenovirus as it is enveloped into the periphery of a cell. Given the contextual situation of the virus, we were able to define that the virus is undergoing the process of endosomal uptake into the cell. The ability to determine the orientation of the virus with respect to the cell membrane allowed the visualization of the position of the adenovirus penton fibre as it interacts with the plasma membrane, being in this instance directly found centrally in the invaginated plasma membrane patch (Fig. 3C, D). The bilayer of the plasma membrane could clearly be followed around the virus in x, y and z, the curvature being indicative of the process of entry being fairly advanced. The immediate proximity of microtubules to the virus undergoing internalization by endocytosis suggests that the subsequent process of viral retrograde transport towards the nucleus is closely coupled to microtubules, consistent with observed speeds for incoming adenoviruses in the range of ∼1 μm s^−1^ (Bremner et al., 2009), which allows the virus to quickly gain entry to the inner regions of the cell.

### 3.4 Evaluation of CTF correction of tomograms for *in situ* sub-volume averaging

Sub-volume averaging of structures from single tomograms is routine until the resolution approaches the first zero of the CTF function for the given defocus at which the tomogram was acquired. In order to achieve accurate information past the first zero, it is important that one efficiently corrects the CTF. In our instance and conditions phase flipping in IMOD was sufficient in a single tomogram to recover significant information contained across the field of view. This is the case despite a large nominal defocus range determined during the acquisition of the tomogram (Table S1). This inaccuracy in the defocus during tomogram acquisition is not uncommon, and the lack of eucentricity across the sample and non-uniformity of the defocus will make the CTF model of individual particles within the tomogram more difficult to determine.

The comparison of microtubule structures reconstructed using CTF and non-CTF corrected tomograms (Fig. 4A and B) for sub-volume averaging from each tomogram clearly shows that even in the case of one tomogram, the resolution to which we can interpret a structure is significant (Fig. 4A). The CTF corrected structure of the microtubule from the U87MG tomogram has an FSC(0.143) of 18.5 Å, and interpretable information persists in the FSC to around 17 Å. Without CTF correction, the amount of information in the structure falls off at around 25 Å (Fig. 4A). In the case of the U2OS tomogram, the non-CTF corrected structure is interpretable to 32 Å due to the CTF induced phase change, but is increased to a resolution of 25 Å after CTF correction (Fig. 4B).

**Fig. 5:**
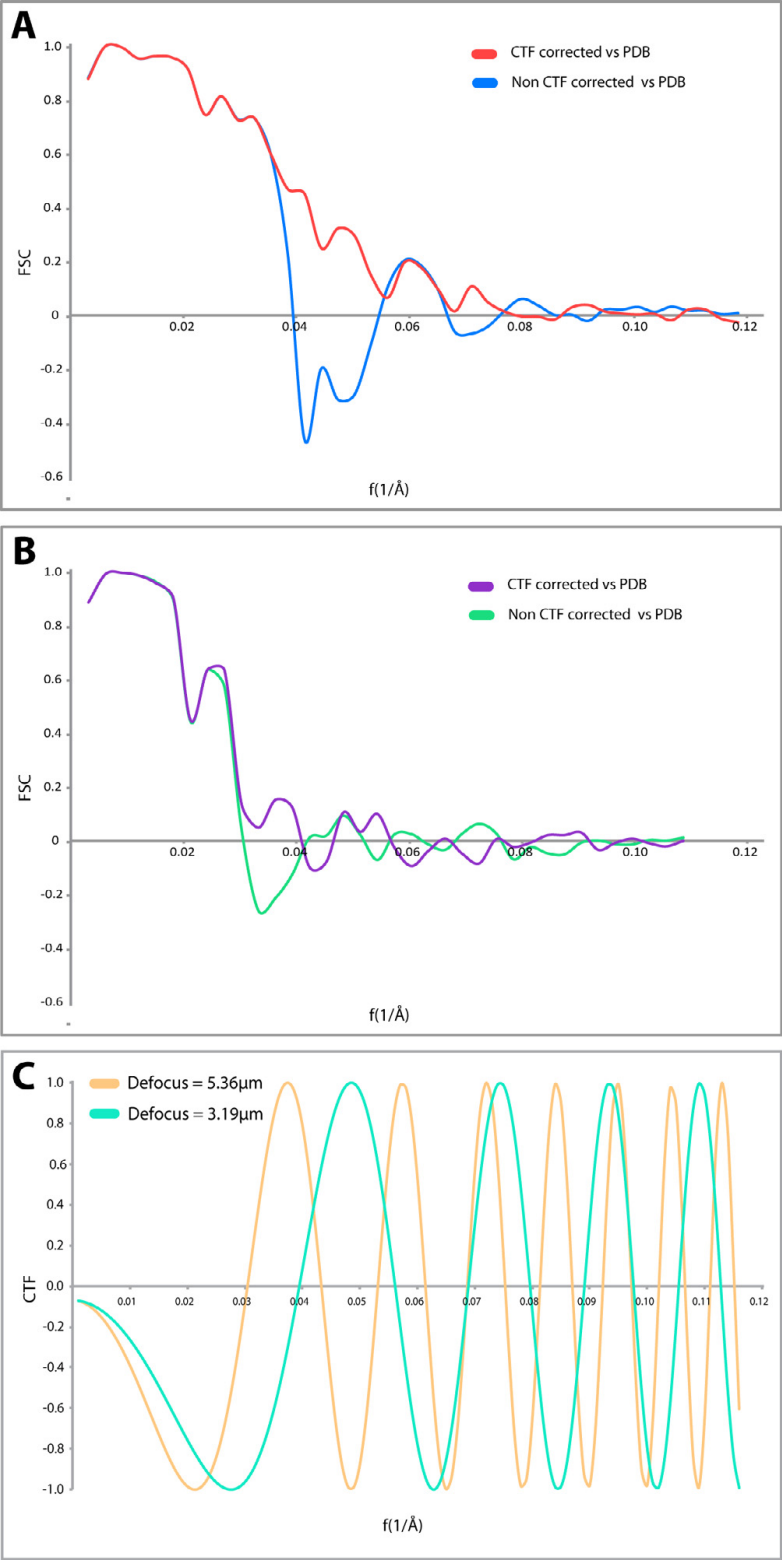
Comparison of sub-volume averaged microtubule structures with a PDB-generated “ideal” 13 protofilament model highlights aberrant points in the structure. The effect of the CTF on elements of the sub-tomogram averaged microtubules structures are shown for A) U87MG and B) U2OS tomograms. A) Shows a pronounced dip below zero between 0.040 and 0.055 corresponding to the theoretical first negative oscillation of the CTF of the microscope, shown in C). An equivalent dip is also visible in B). A negative dip below 0 in the correlation of the CTF-corrected structure may indicate an inaccuracy in defocus determination (c.f. Fig. 4).

Interestingly, the calculation of the defocus in IMOD of each tomogram was different to the nominal defocus of the microscope by as much as 0.5 μm (Table S1). Even accounting for inaccuracies in defocus determination of the microscope (Danev and Baumeister, 2016), this is still a large discrepancy. The calculated CTF of the calculated defoci is shown in Fig. 4C for each tomogram. To better determine the accuracy of the CTF correction, an ‘ideal’ microtubule structure was built by fitting atomic models of symmetrized tubulin monomers from a microtubule structure (PDB ID: 3J2U) into the sub-tomogram averages determined here. These atomic models were then Gaussian blurred in Chimera to Nyquist frequency and matched in sampling and box size to the respective sub-tomogram average. The FSCs between this ‘ideal’ structure and the CTF and non-CTF corrected averages are shown in Fig. 5. For both structures, CTF correction resulted in a more accurate structure, with the U87MG cell microtubule average (Fig. 4A) displaying a particularly obvious phase flip correction. However, a small dip below zero at around 25 Å in the U2OS cell microtubule average CTF-corrected FSC (Fig. 4B, purple line) may well indicate an inaccuracy in defocus determination.

We would suggest that an “all-tomogram” approach to CTF correction, especially for datasets with low particle numbers, likely results in corrections that are less accurate or not weighted correctly. Approaches that have been implemented recently (Bharat et al., 2015, Galaz-Montoya et al., 2016) whereby individual sub-volumes are tilt-dependently CTF corrected should prove to be much more accurate in determining a per-particle CTF correction. These approaches would yield a much more accurate determination of particle eucentricity across the tilt series, as a custom per-particle defocus and z-position within the tomogram can be determined. This should result in sub-volume averages that yield better results, particularly for single tomograms, or datasets with only a low number of particles.

### 3.5 Determination of virus structure *in* situ improves understanding of virus-host interactions

The current structural understanding of virus-host interactions stem mainly from studies using co-crystalized complexes, small angle scattering and sub-volume averaging of *in vitro* constituted systems, such as virus-like particles. Direct molecular imaging of the viral interactions with the host has hitherto not been a mainstay of investigation.

The increased signal-to-noise ratio in the images gained from the use of direct detection allowed us to see much greater details inside the cell, as well as to average structures to an intermediate resolution capable of bridging the gap between structural context and molecular resolution (Fig. 2, Fig. 3). This will surely allow us to provide novel insights into the cellular biology of viral infection. The structural context of virus-cell processes in a model cell line is currently the closest one may currently come to reconstituting a disease-like state. The ability to localize structures in their cellular context (Fig. 3), and to sub-volume average different conformers of the virus as it progresses through various stages of its life cycle (Fig. 3C), will allow determining which viral proteins are lost, which cellular proteins it interacts with, and what other biological macromolecules and pathways the virus recruits or hijacks in this dynamic process. Being often easily recognizable due to their unique structural signatures viruses also constitute ideal tools to probe cellular processes. Furthermore, the structural determination of cellular factors co-localising with the virus during infection gives clues to potential novel forms of interventions to be explored.

### 3.6 Challenges and perspectives in the cryoET of cells

Many biological phenomena are intrinsically transient, and rare. Structural biology currently relies on approaches that can enrich for a particular biological event, state, or process of biological relevance, e.g. by overexpression of a particular protein, though this often comes with the sacrifice that the system is no longer near native. These are typified by the approaches based on purified material and can be combined with X-ray crystallography, single particle cryoEM or sub-tomographic averaging. However, these necessary and, in many cases sensible approaches, cannot always be applied due to the difficulty of producing substantial amounts of protein, DNA/RNA or other macromolecule in a structurally determinable state. Furthermore, the lack of *in situ* context for these structures means that in many cases crucial pieces of information from the overall model of how a biological process occurs are missing, such severely limiting a mechanistic understanding.

In this paper, we have discussed methods and data that allow for assessing the wealth of detail that can be mined from a single tomogram. Resolutions of 77 Å from a single symmetrized icosahedral virus and 18.5–25 Å from relatively few numbers of microtubule segments gives us a good indication that in many cases, the amount of information present even in a single tomograms is substantial, and something that, given the right biological problem and system, may lead to novel insights to complement the structural models many laboratories are building using canonical techniques.

The direct observation of biology just at the cryoET accessible periphery of a cell provides a severe limitation; as the feature of interest needs to reside in an area thin enough to image with sufficient counts. This limits the range of biological processes one can image and the number and quality of observed features. Accordingly, several approaches have been implemented addressing this shortfall by not only making localization of a biological feature of interest easier to find, but also adding to the overall interpretation of the cryoET data (Schellenberger et al., 2014, Schorb and Briggs, 2014). The recent success provided by the use of thinning methods (see below) is another promising direction.

Recent developments in phase plate technologies and in cryoFIBSEM (Asano et al., 2015, Danev et al., 2014, Mahamid et al., 2016, Rigort et al., 2012) have given a flavor of the insights that may be gained in the future, specifically with the ability to investigate by cryoET processes and structures deeper inside a cell, and e.g. probe nuclear biology in its native context. However, these applications are currently still not routine or for higher throughput uses, and require specialist knowledge for their operation and application. These current drawbacks are significant, as they restrict the range of biological applications that may benefit from the improved insight that could be determined. Future developments need to address these, and make the technologies much easier to use and robust for routine everyday use.

A potentially powerful technique to provide further context for *in situ-*determined viral structure is the coupled use of correlative light electron microscopy (CLEM). Techniques such as light sheet fluorescence microscopy (LSFM), 3-dimensional structured illumination microscopy (3D-SIM) and localization microscopy (Chen et al., 2014, Schermelleh et al., 2008), especially when utilized in combination (Hoyer et al., 2016) offer potential for coupling the many complimentary techniques for cryoET, such as CLEM and cryoFIB/SEM, to offer a multi-scale resolution view of a molecular process *in situ* (Mahamid et al., 2015). Performing super-resolution fluorescence microscopy modalities routinely under cryo conditions and ultimately in a CLEM fashion (cryoCLEM) is the next challenge, but the few pilot studies performed demonstrate the potential and feasibility of such an approach (Wolff et al., 2016).

The examples highlighted in our study suggest that it is possible to gain valuable structural insight from only few tomograms, and a 25 Å 3D structure should be possible from fewer than 300 particles. Subsequent classification, back plotting into the tomogram, and segmentation can provide a diverse range of biological information, in the most native of contexts, over a wide range of resolutions.

The work presented in this article provide a significant and exciting frontier in cellular biology that may one day lead to the structural determination of novel structural complexes *in situ*. The multitude of classical electron microscopy applications many of which could be imaged under the much more native environment provided by cryoET, promise a rich and plentiful vein of discovery that can be judiciously mined through appropriate fluorescently tagged biological systems. This link between the high resolution imaging of cellular structure and structure determination is so far poorly explored and advancements would lead to a wealth of novel biological insight.

## 4 Concluding remarks

CryoET of cells offers a genuine route to the structural determination of subcellular complexes and organelles in their native state in the course of their function, and at a resolution gradually closer approaching that of single particle analysis. Improvements in microscopic hardware, software workflows, sample preparation and transfer, and computational processing will require future investment if cryoET is to realize its full potential.

## 5 Accession codes

The microtubule sub-volume average reconstructions have been deposited in the Electron Microscopy Data Bank (EMDB) at PDBe (http://www.ebi.ac.uk/pdbe/emdb/) as EMD-4043 and EMD-4045.
