# Supplementary material for: Cellular electron cryo tomography and in situ sub-volume averaging reveal the context of microtubule-based processes
Source: J Struct Biol. 2017 Feb;197(2):181–90. doi: 10.1016/j.jsb.2016.06.024 (PMC5287354; doi:10.1016/j.jsb.2016.06.024)
Supplement: Supplementary Figures S1–S6 and Table S1 [file mmc1.doc]

**Supplementary Material**

Supplemental Figures

**Figure S1** – Tomographic slices in 3-dimensions (XY, XZ and YZ) of U87MG tomogram used for analyses in Figure 1 and 2. Inset shows the position of XZ (blue line) and YZ (green line) in relation to the XY slice. Scale = 100nm


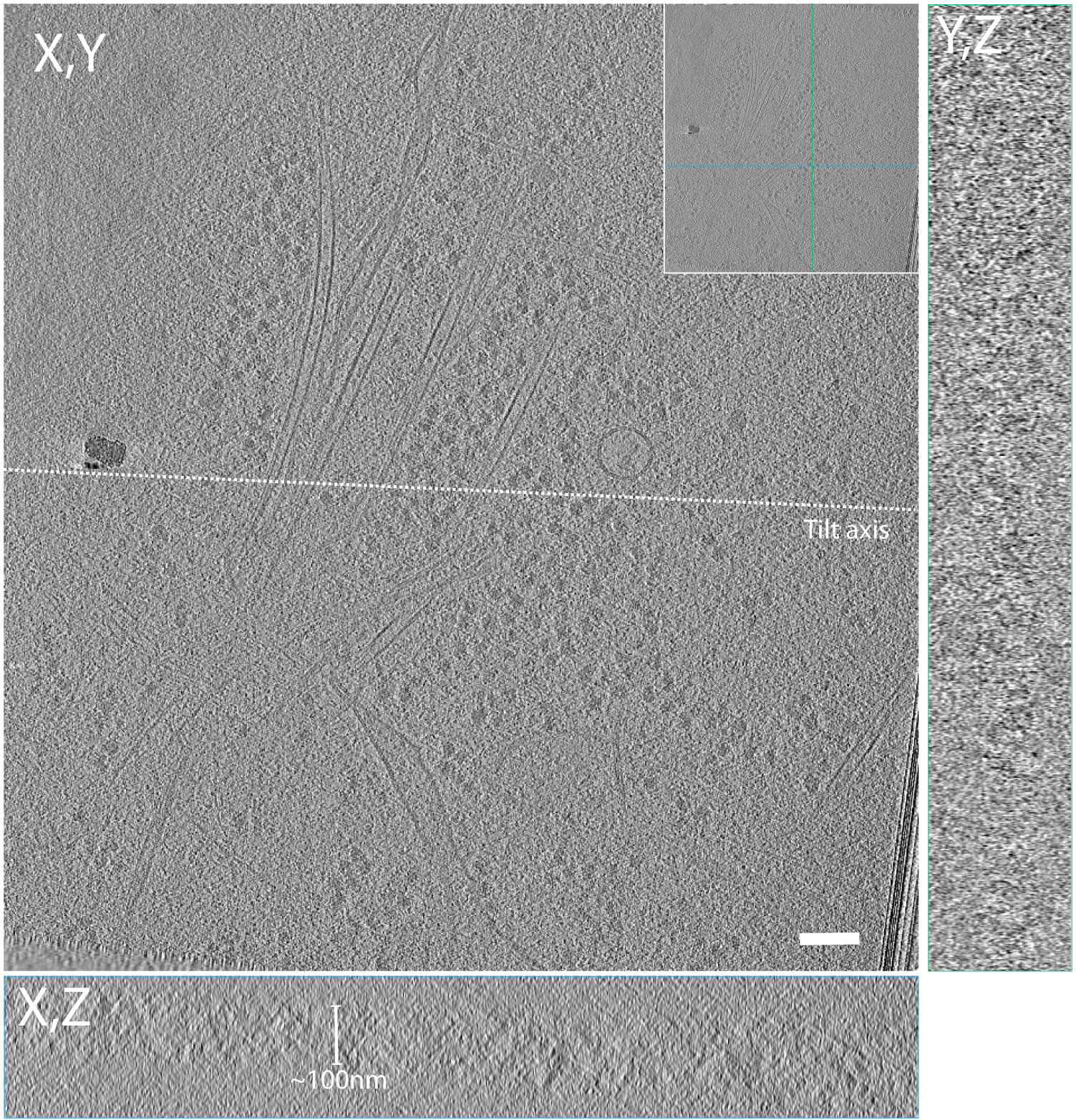


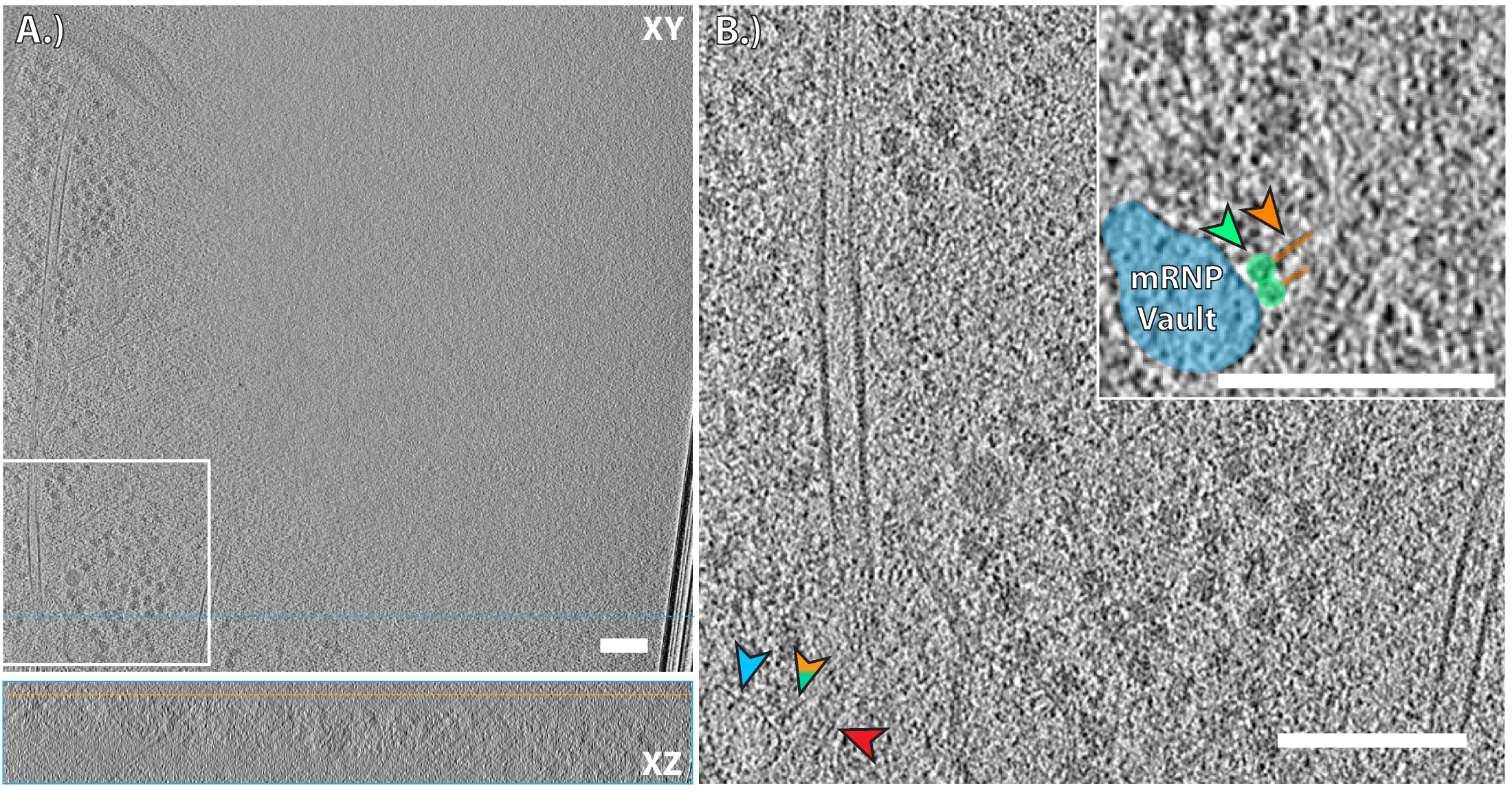


**Figure S2** – Structural context of Vault protein in relation to the tomographic volume shown in Figure 1. (A) Box outlined by a dashed line in the XY slice (along the orange line in XZ; XZ sliced along the blue line XY) indicates the position of vault within the tomogram. (B) zoom into area inside the white box in (A). The blue arrow and area (inset) highlights the position of the ribonucleoprotein Vault, the green/orange arrows highlight the dynein dimer (green = AAA+ domain and orange = stalk domain) and the red arrow highlights the top of the microtubule in contact with the dynein dimer. Scale bars = 100nm.

**Figure S3** – Tomographic slices in 3-dimensions (XY, XZ and YZ) of U2OS tomogram used for analysis in Figure 3. Inset shows the position of XZ (blue line) and YZ (green line) in relation to the XY slice. Yellow lines indicate the thickness of the ice. Scale = 100nm.


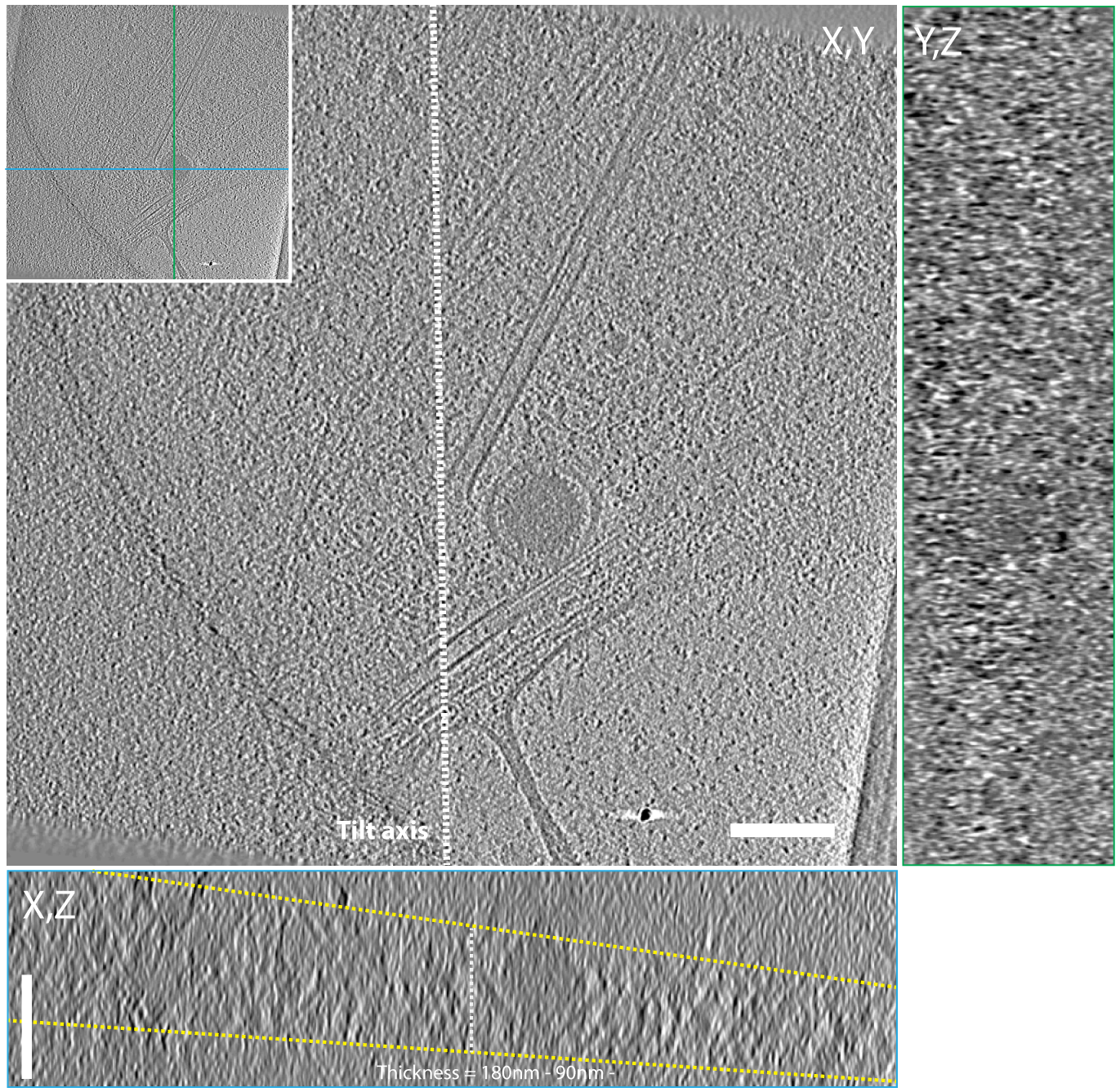

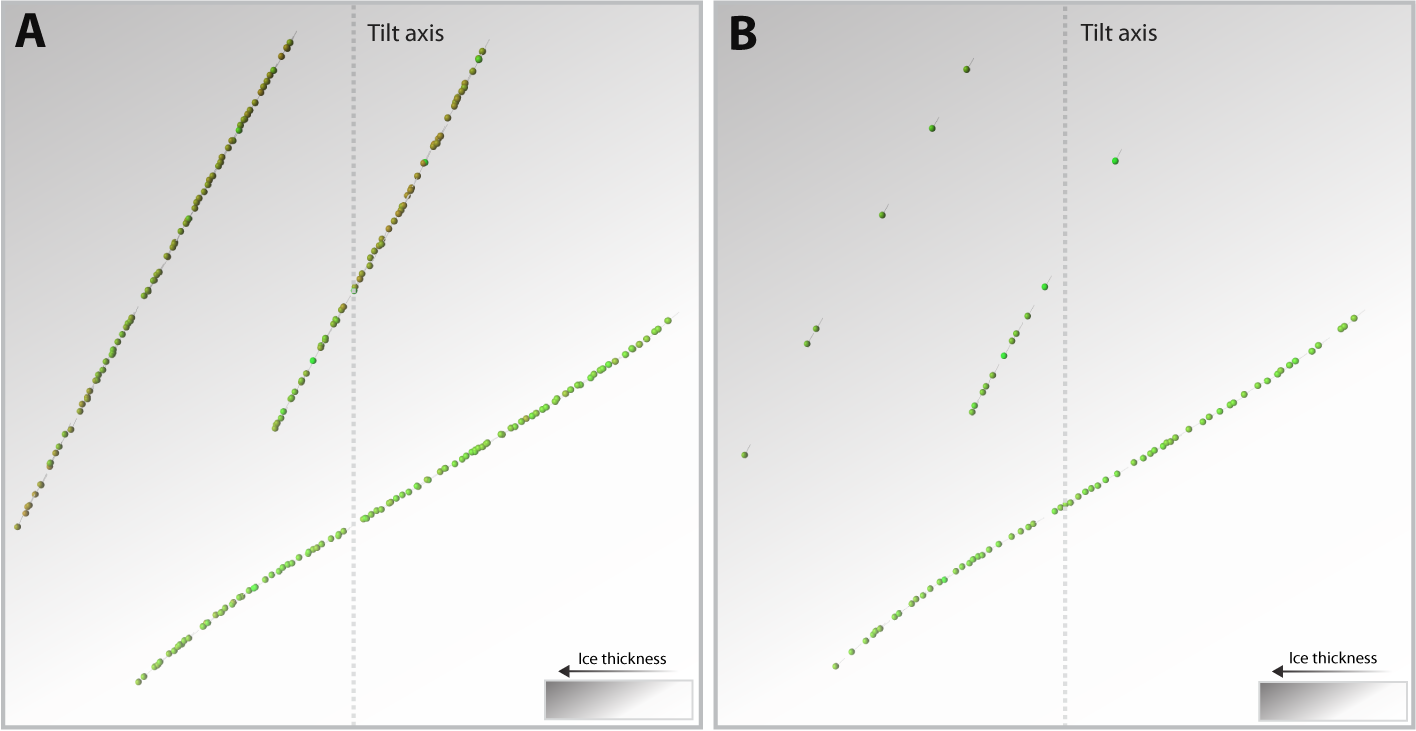


**Figure S4** – Model of the linear model used to determine the microtubule structure in Figure 3. **A)** Orange/green spheres determine the position relative to the tilt axis of sub-volumes with high (green) and low (orange) cross correlation to the average. **B)** Model of sub-volumes after removal with a cross correlation of 0.2. Black and white gradient in both **A** and **B** approximated the thickness gradient across the tomogram.

**Figure S5** – Comparison of “gold standard” FSC curves for CTF and non-CTF determined structures for **A)** U87MG tomogram and **B)** U2OS tomogram.


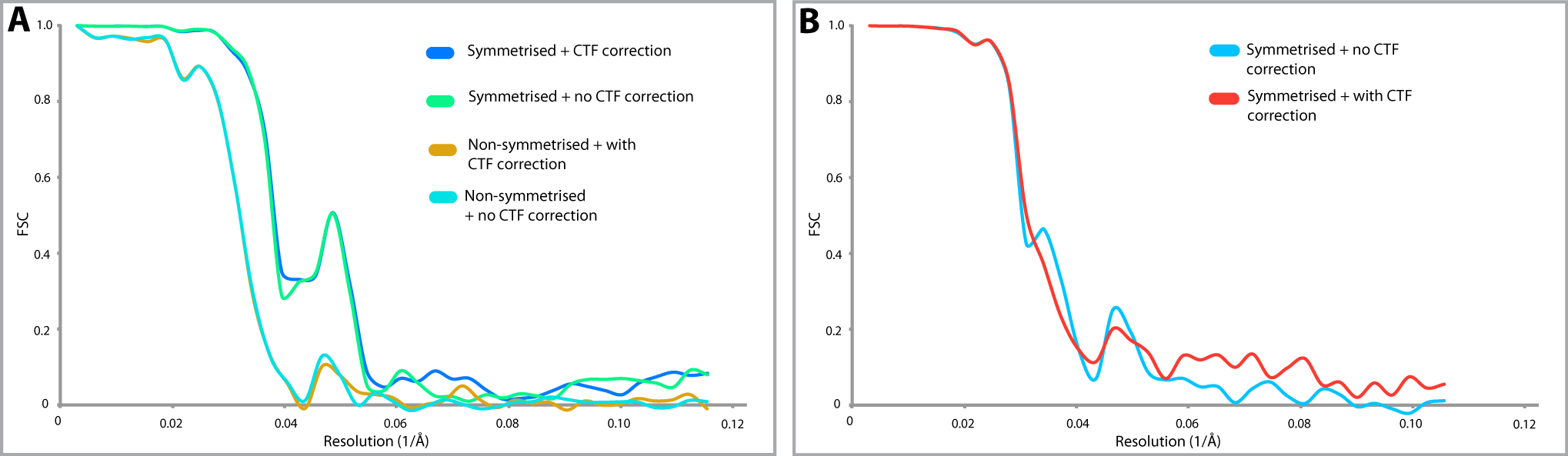

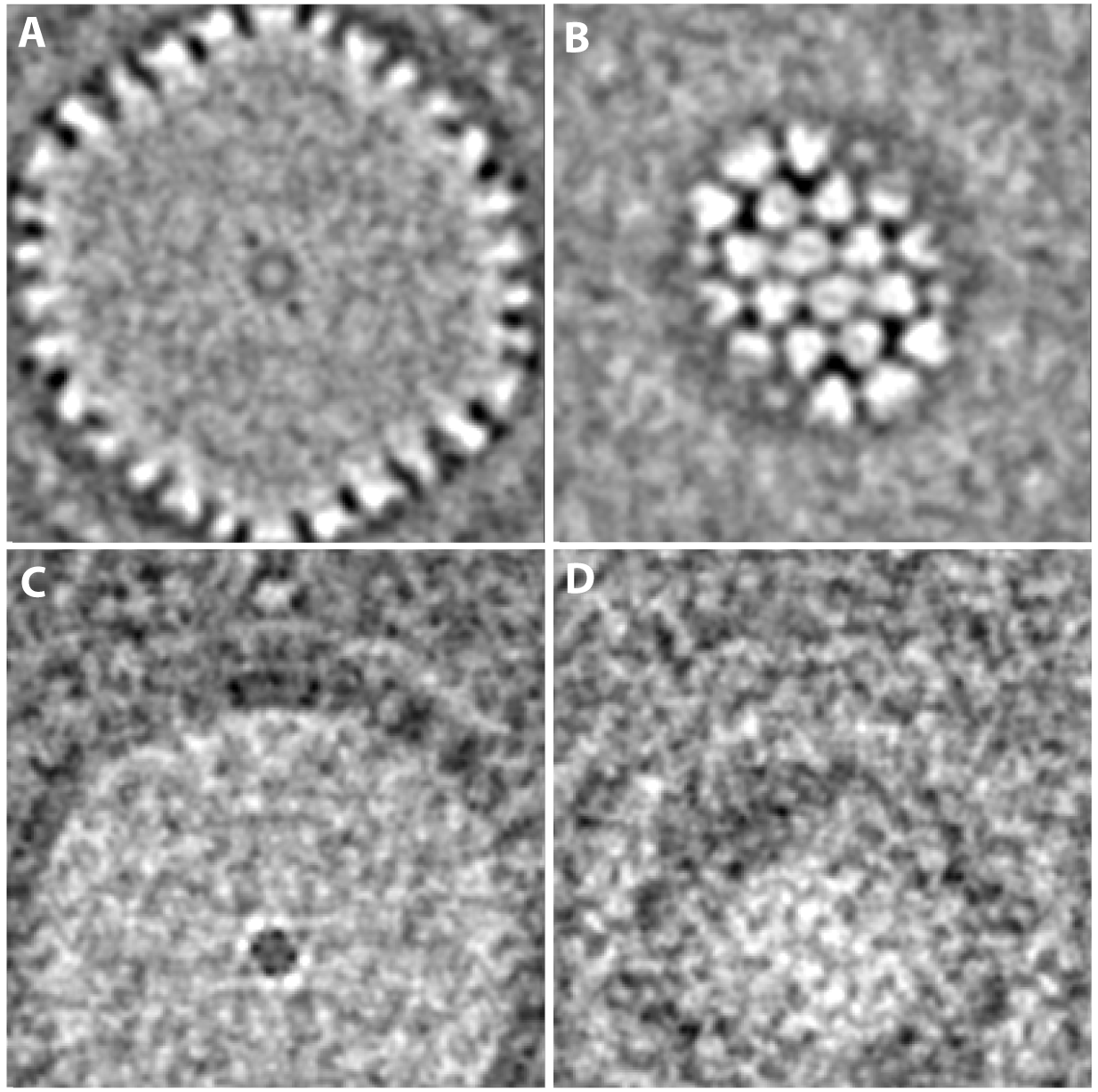


**Figure S6** – Symmetrized structure of the Adenovirus in the U2OS tomogram. **A)** and **B)** shows the middle and top slice, respectively, of the adenovirus structure shown in Figure 3 after symmetrization of the virus using the refined Euler angles resulting from an orientation search for EMDB-1574 filtered to 20Å. **C)** and **D)** shows the middle and top slice, respectively, prior to any refinement, rotationally or translationally, against EMDB-1574.

| **Tomogram:** | **U87MG Cell** | **U2OS Cell** |
| --- | --- | --- |
| Nominal microscope defocus (µm) | -5.0 | -4.0 |
| Reported defocus range in acquisition (µm) | -5.13 to -4.57 | -4.16 to -3.49 |
| Calculated defocus (µm) | -3.19 | -5.39 |
| Total Dose (eÅ^-2^) | 72.4 | 75.2 |
| No. particles before symmetrisation | 312 | 148 |
| No. particles after symmetrisation | 4056 | 1924 |
| Tilt range (degrees) | -57 to +60 | -45 to +45 |
| Tilt sampling (degrees) | 3 | 3 |
| Calibrated magnification | 11848x | 21739x |
| Calibrated pixel size of average (Å) | 4.22 | 4.6 (2.3 unbinned) |
| Approximate ice thickness (nm) | 80-100 | 150-180 |

**Table S1** – Table of parameters used for acquisition and sub-volume averaging of the microtubules from the U87MG and U2OS cell lines.
